# Differential Insulin Secretion of High-Fat Diet-Fed C57BL/6NN and C57BL/6NJ Mice: Implications of Mixed Genetic Background in Metabolic Studies

**DOI:** 10.1371/journal.pone.0159165

**Published:** 2016-07-12

**Authors:** Camille Attané, Marie-Line Peyot, Roxane Lussier, Dongwei Zhang, Erik Joly, S. R. Murthy Madiraju, Marc Prentki

**Affiliations:** Departments of Nutrition and Biochemistry, Montreal Diabetes Research Center, CRCHUM and Université de Montréal, Montréal, QC, Canada; Centre National de la Recherche Scientifique, FRANCE

## Abstract

Many metabolic studies employ tissue-specific gene knockout mice, which requires breeding of floxed gene mice, available mostly on C57BL/6N (NN) genetic background, with cre or Flp recombinase-expressing mice, available on C57BL/6J (JJ) background, resulting in the generation of mixed C57BL/6NJ (NJ) genetic background mice. Recent awareness of many genetic differences between NN and JJ strains including the deletion of nicotinamide nucleotide transhydrogenase (*nnt*), necessitates examination of the consequence of mixed NJ background on glucose tolerance, beta cell function and other metabolic parameters. Male mice with NN and NJ genetic background were fed with normal or high fat diets (HFD) for 12 weeks and glucose and insulin homeostasis were studied. Genotype had no effect on body weight and food intake in mice fed normal or high fat diets. Insulinemia in the fed and fasted states and after a glucose challenge was lower in HFD-fed NJ mice, even though their glycemia and insulin sensitivity were similar to NN mice. NJ mice showed mild glucose intolerance. Moreover, glucose- but not KCl-stimulated insulin secretion in isolated islets was decreased in HFD-fed NJ vs NN mice without changes in insulin content and beta cell mass. Under normal diet, besides reduced fed insulinemia, NN and NJ mice presented similar metabolic parameters. However, HFD-fed NJ mice displayed lower fed and fasted insulinemia and glucose-induced insulin secretion *in vivo* and *ex vivo*, as compared to NN mice. These results strongly caution against using unmatched mixed genetic background C57BL/6 mice for comparisons, particularly under HFD conditions.

## Introduction

Genetically modified mice using Cre recombinase/loxP system are extensively used to perform conditional gene deletion experiments to assess molecular mechanisms involved in the etiology of type 2 diabetes (T2D) [1] as well as in many other diseases. This requires mice with targeted allele (i.e., floxed gene) for a specific gene to be bred with transgenic mice expressing Cre-recombinase enzyme under the control of tissue specific promoters. Most of the floxed mice, for a variety of genes, have been generated using targeted embryonic stem (ES) cells from the International Knockout Mouse Consortium (IKMC). IKMC used ES cells from C57BL/6N (NN) mice to generate targeted alleles and mice. The final production of floxed genes requires breeding of these mice carrying targeted alleles with Flpo recombinase transgenic mice and floxed mice are then mated with Cre recombinase transgenic mice. As most of the available Cre or Flpo recombinase transgenic mice are on C57BL/6J (JJ) background [2], crossing these mice results in animals having mixed NJ background, which are then mated together to generate the control floxed allele and tissue-specific knockout (KO) mice. Thus, the mice generated this way present different C57BL/6 genetic backgrounds (NN, JJ and NJ) within each group (control and KO mice). The vast majority of studies did not specify the substrains of the mice that were used in the metabolic experiments using the C57BL/6 strain [2]. In such selections, the possibility that mouse strain may have a significant impact on glucose homeostasis and β-cell function [3–5] was not considered. Indeed, these differences became evident in studies using RIP (rat insulin promoter)-Cre mice. Lee and colleagues reported that RIP-Cre mice on different genetic backgrounds (C57BL/6:129, C57BL/6N and C57BL/6J) from three laboratories displayed impaired glucose tolerance due to altered insulin secretion [6], whereas normal glucose tolerance and insulin secretion was reported when using RIP-Cre mice on a pure genetic background [7]. Thus, confounding findings can be obtained when mice on a mixed background are used.

The C57BL/6 strain is a widely used model for diet-induced obesity because of its high susceptibility to develop obesity and hyperglycemia when fed with a high-fat diet compared to other strains [8,9]. C57BL/6 mice were originally developed in 1921 at the Bussey Institute (Harvard University) and colonies of these mice were maintained in different labs throughout the world, which gave rise to several substrains [2]. These different substrains, particularly JJ and NN, which are the most commonly used substrains in metabolic studies, display many genetic and phenotypic differences [2].

Some studies reported that JJ mice display glucose intolerance and reduced insulin secretion when compared to other mouse strains (DBA/2, C3H/HeJ and AKR/J) [3,9–12] or NN mice [13–16] even though these results were not confirmed by others [17,18]. Similarly, the response to High-Fat Diet (HFD) is different between different C57BL/6 substrains, and the results were contradictory among different labs [14,19–21]. Mouse substrains also differ in behavior [22,23], alcohol responsiveness [24], as well as susceptibility to tumor formation [25].

Among the several genetic differences identified between JJ and NN substrains, deletion of Nicotinamide Nucleotide Transhydrogenase (NNT) gene has been addressed by many laboratories. NNT, a mitochondrial enzyme that catalyzes the reversible transfer of reducing equivalents from NADH to NADP [26], has been considered as a plausible genetic change to explain glucose intolerance and defective insulin secretion observed in JJ mice [12], which harbor a spontaneous in-frame 5-exon deletion in the NNT gene with a complete loss of the protein [12,27]. Only the JJ substrain from the Jackson Laboratory has the NNT mutation but not the NN strain supplied by Taconic or Charles River [15]. A positive correlation between NNT activity and first-phase insulin secretion [8,12] and phenotype rescue of JJ mice by transgenic expression of NNT suggested a role for this enzyme in the control of insulin secretion and its loss as a possible reason for the observed glucose intolerance and impaired insulin secretion in JJ mice [15]. Besides the NNT mutation, 36 SNPs and small indels as well as 15 structural variants, including a copy number variation for the insulin degrading enzyme gene [28], were found to be different between NN and JJ strains [16] and likely contribute to the phenotypic differences. The overall impact of each of these genetic variances between NN and JJ mice on their phenotypic differences remains to be assessed.

In light of the phenotypic and genotypic differences between NN and JJ mice, a growing number of studies cautioned regarding the use of mice on a mixed genetic background [2,27,29]. As mentioned above, breeding of different C57BL/6 substrains (NN and JJ) results in the generation of both NN, NJ (mixed genetic background) and JJ mice and mis-pairing these C57BL/6 substrains for making control and tissue-specific KO mice can lead to variability and often discrepant results [2,29]. While JJ mice displayed a defective insulin secretion versus NN mice [8,12,15], the phenotype of NJ mice in comparison with NN mice, and the potential loopholes in using these NN and NJ mice disregarding their genotype, has never been studied to our knowledge. In the present study, we addressed this potential problem of working on a mixed C57BL/6 background, by examining the metabolic responses of NJ and NN littermate mice in physiological context (chow diet) and in metabolic stress condition (HFD).

The results indicated that although the NJ genotype of mice had no effect on body weight and food intake, insulinemia under fed and fasted conditions and after glucose challenge in HFD-fed mice were decreased in association with mild glucose intolerance without changes in fed/fasted glycemia. Moreover, *ex vivo* glucose-stimulated insulin secretion was also decreased in NJ mouse pancreatic islets compared to NN mice. Thus, caution must be exercised when using C57BL/6 mice having mixed background as results from such studies can be potentially misleading.

## Material and Methods

### Materials

Glucose-free RPMI 1640 media was purchased from Gibco (Burlington, ON, Canada). Fatty acid free BSA and all chemicals, unless otherwise specified, were purchased from Sigma-Aldrich (St-Louis, MO, USA).

### Animals

Initially, homozygous ATGL flox/flox (fl/fl) mice on a JJ background [30] were crossed with heterozygous MIP-Cre-ERT (Mcre) mice on a NN background [31]. Heterozygous mice obtained in the F1 generation were bred to produce wild-type (WT), MCre and fl/fl mice on NN, NJ or JJ background (F2 generation). MCre and WT on a NN or NJ background were used for oral glucose tolerance test.

For other experiments, fl/fl mice heterozygous for the NNT mutation (NJ) were crossed with fl/fl mice expressing the wild-type NNT allele (NN) to generate wild-type (NN) or heterozygous (NJ) NNT allele. PCR was performed on offspring tail DNA to distinguish among wild-type or mutant NNT alleles as described previously [14]. Male mice were housed 3–4 per cage on a 12 h light/dark cycle at 21°C with free access to water and standard diet (ND; normal diet, Teklad Global 18% protein rodent diet; Harlan Teklad, Madison, WI, 15% fat by energy). For feeding experiments, 11-week-old male mice were placed in individual cages and were fed with either ND or HFD (Bio-Ser Diet #F3282, Frenchtown, NJ, 60% fat by energy). Body weight and energy intake were measured weekly. After 12 weeks on HFD, mice were anesthetized with ketamine/xylazine administered by intraperitoneal injection. After confirmation of the anesthesia by lack of responsiveness to toe pinching, blood was collected by cardiac puncture. Animals were then sacrificed by cervical dislocation and pancreas was collected for beta-cell mass analysis or was injected by collagenase to isolate islets. All procedures were approved by the Institutional Committee for the Protection of Animals at the Centre Hospitalier de l’Université de Montréal.

### Plasma parameters

Blood glucose was determined by a portable glucometer (Accu-check Advantage, Roche, Indianapolis, IN). Blood was collected between 8:00 and 10:00 am in fed or overnight fasted mice. Plasma insulin was measured by ELISA (UltraSensitive mouse Insulin ELISA Kit, Alpco Diagnostics).

### Oral Glucose Tolerance test (OGTT)

OGTT was performed in 19-week-old mice fed either standard or HFD. Glucose (2g/kg body weight) was administered orally by gavage in conscious mice in the morning after a 16 h fasting. Tail blood glucose was measured at 0-15-30-60-90 and 120 min after glucose administration, using a glucometer, and the blood samples were also processed to quantify insulinemia (UltraSensitive mouse Insulin ELISA Kit, Alpco Diagnostics).

### Insulin Tolerance test (ITT)

ITT was performed in 21-week-old mice fed a HFD. Human recombinant insulin (Eli-Lilly, Indianapolis, IN; 0.75 units/kg body weight) was injected intraperitoneally in conscious mice at 2:00 pm after 4-h food withdrawal. Blood glucose was measured at 0-15-30-45-60-90 and 120 min after insulin administration using a glucometer.

### Insulin secretion *ex-vivo*

Islets from 23-week-old NN or NJ mice fed a HFD were isolated as described previously [32]. Immediately after isolation, islets were distributed in 12-well plates (10 islets/well) in RPMI 1640 medium containing 3 mM glucose and kept at 37°C for 2 h followed by preincubation for 45 min at 37°C in Krebs Ringer Bicarbonate (KRB) medium with 10 mM Hepes (KRBH) containing 0.5% defatted-BSA and 3 mM glucose. Islets were then incubated for 1 h at 3, 8, or 16 mM glucose, in the presence or absence of palmitate/oleate (0.15mM each) in KRBH, 0.5% defatted-BSA, and also at 3 mM glucose plus 35 mM KCl. At the end of the incubations, insulin in the media and islet insulin contents were quantified using AlphaLISA insulin immunoassay kit (Perkin Elmer, Waltham, MA) and human insulin as standard.

### Beta cell mass

Beta cell mass was determined as previously described [13].

### Statistical analysis

Results are expressed as means ± SEM. Statistical significance was calculated with the Student’s unpaired two-tailed *t*-test or two-way analysis of variance (ANOVA) with Bonferroni post hoc test for multiple comparisons, as indicated, using the GraphPad Prism software version 6.0. A *p* value ≤0.05 was considered significant.

## Results

We first realized the problems with the use of mixed genetic background mice during our studies on β-cell specific adipose triglyceride lipase (ATGL)-KO mice (Attané et al, unpublished data). In order to generate β-cell specific ATGL-KO mice, we first mated ATGL fl/fl mice on a JJ background with MipCre-ERT mice on a NN background. Mice from the F1 generation, on a NJ background, were then mated together to produce the β-cell specific ATGL KO mice. This breeding strategy resulted in mice having NN, NJ or JJ background in the same litter. Considering that isolated islets from JJ mice are known to have insulin secretion defect compared to NN mice, we decided to examine whether there is any impact of the heterozygous NJ background on metabolic parameters, which has never been studied. Thus, we assessed the effect of heterozygous NJ background on whole body energy homeostasis and insulin secretion in comparison to NN mice to better understand the impact of mixed genetic background.

### NN versus NJ genotype has no effect on body weight and food intake in mice

**Fig 1:**
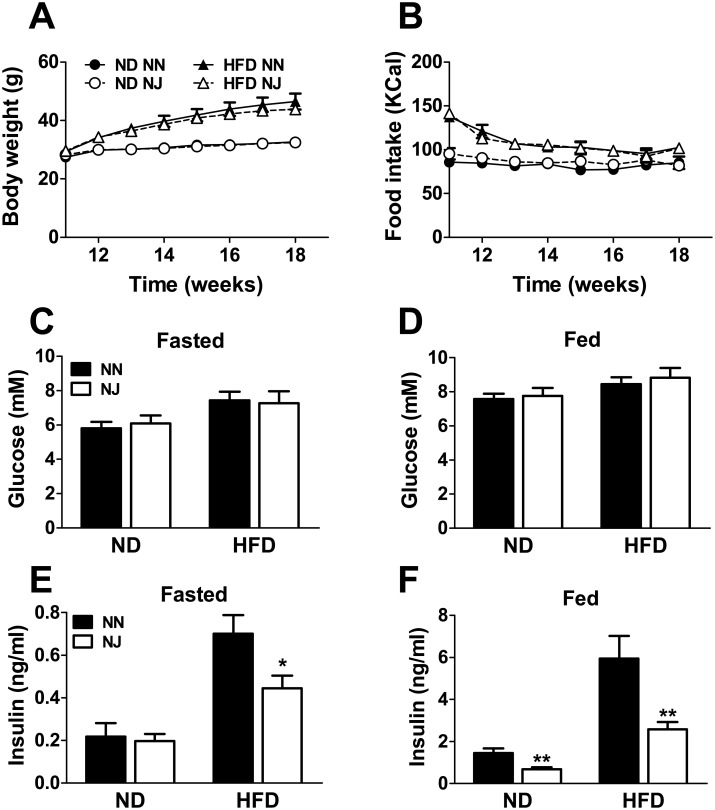
Body weight, food intake, glycemia and insulinemia in ND- and HFD-fed NN and NJ mice. Body weight (A) and food intake (B). Glycemia and insulinemia in overnight fasted (C and E) or fed (D and F) mice. ND, normal diet; HFD, high fat diet. Results are means ± SEM of 7–9 mice in 2–3 independent experiments. *p<0.05 and **p<0.01 compared to NN mice (Student’s t-test).

Mice on C57BL/6N background (NN) as well as on mixed C57BL/6NJ background (NJ) were fed with either a normal or a high fat diet for a period of 12 weeks. Body weight (Fig 1A) and food intake (Fig 1B) were similar in NN and NJ mice fed either HFD or ND.

### Genotype dependent effects on insulinemia but not on glycemia

There was no effect of NJ background on glycemia under fasted and fed (Fig 1C and 1D) conditions, with either diet. However, insulinemia under fed and fasted state was lower in NJ mice on both ND and HFD conditions, than in NN mice (Fig 1E and 1F). These results suggested that insulin secretion response is different in mixed genetic background NJ compared to NN mice.

### Oral glucose and insulin tolerance tests in NN and NJ mice

**Fig 2:**
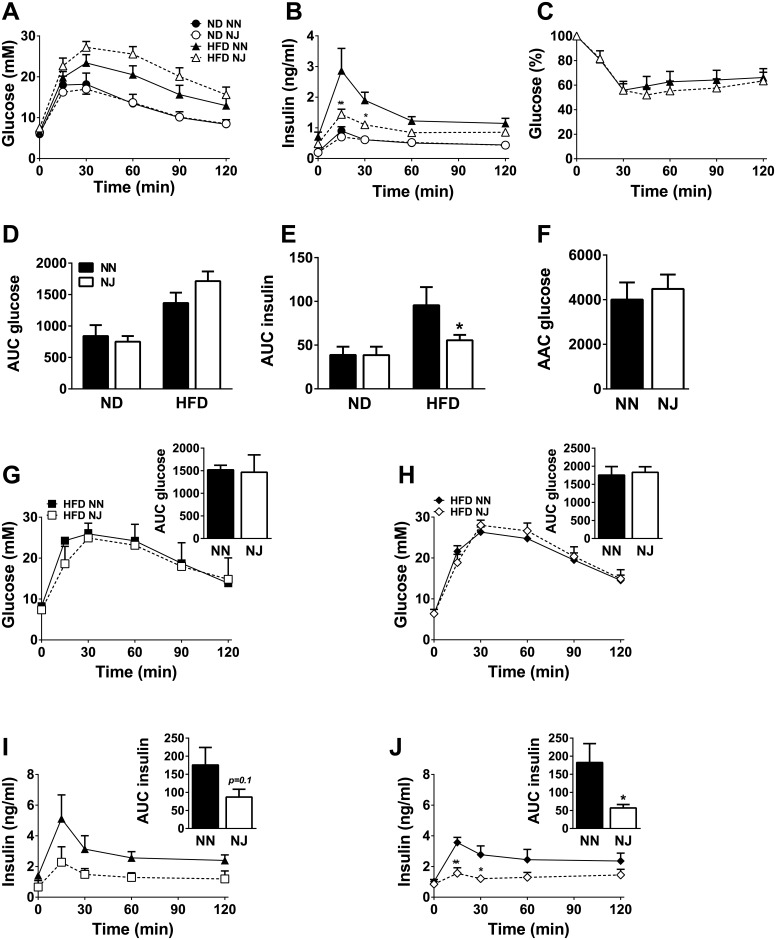
OGTT and ITT in NN and NJ mice. Glycemia **(A)** and insulinemia **(B)** were measured after glucose administration at time 0 in ND or HFD-fed NN and NJ mice and area under the curve (AUC) was calculated for glycemia **(D)** and insulinemia **(E)** curves. Glycemia during ITT and area above the curve (AAC) **(C and F)** in NN or NJ mice fed a HFD. Results are means ± SEM of 7–9 mice in 2–3 independent experiments. Glycemia was also measured after glucose administration in HFD-fed NN and NJ WT **(G)** or MCre **(H)** mice. In the same OGTT tests, insulinemia was measured in NN and NJ WT **(I)** or MCre **(J)** mice. Insets depict AUC for glycemia and insulinemia curves. Results are means ± SEM of 3 WT and 6 MCre mice/group in 2–3 independent experiments. *p<0.05 and **p<0.01 compared to NN mice under the same diet (two-way ANOVA and Bonferroni post hoc test or Student’s t-test).

In order to ascertain whether the lower insulinemia seen in NJ mice is indeed due to lower glucose-stimulated insulin secretion *in vivo*, insulin response to glucose was assessed by OGTT. NN and NJ male on ND displayed similar glucose tolerance (Fig 2A and 2D) and insulinemia (Fig 2B and 2E) after oral glucose challenge. Glucose tolerance was markedly decreased as expected after HFD feeding (Fig 2A and 2D). HFD fed NJ mice showed mild glucose intolerance with slightly higher glycemia response than NN mice, even though the results were not statistically significant (Fig 2A and 2D). In HFD fed mice, insulinemia during the OGTT was elevated in both the genotypes, as expected. However, glucose-induced insulin secretion was 40% lower in HFD fed NJ mice in comparison to NN mice (Fig 2B and 2E). We then examined whether the reduced insulinemia in fed and fasted state as well as after a glucose challenge during OGTT observed in NJ mice without major changes in glycemia is related to altered insulin sensitivity. Insulin tolerance test revealed similar effect of insulin on glycemia in HFD fed NN and NJ (Fig 2C and 2F) mice, indicating that insulin sensitivity was not different between the genotypes.

Finally, to discount the possibility that the decreased insulinemia during OGTT in NJ mice might be related to the presence of ATGL floxed alleles, an OGTT was also done in HFD-fed WT and MCre mice on a NN or NJ background. Despite no change in glucose tolerance in both WT and MCre NJ mice (Fig 2G and 2H, respectively), insulinemia was decreased after the glucose challenge in WT and MCre NJ mice (Fig 2I and 2J, respectively), as we observed in fl/fl mice. Altogether, these results confirmed that the insulin secretion defect observed in NJ mice is due to the mixed background of these mice and is present in all experimental groups (WT, MCre and fl/fl).

### Insulin secretion in isolated islets from HFD-fed NN and NJ mice

**Fig 3:**
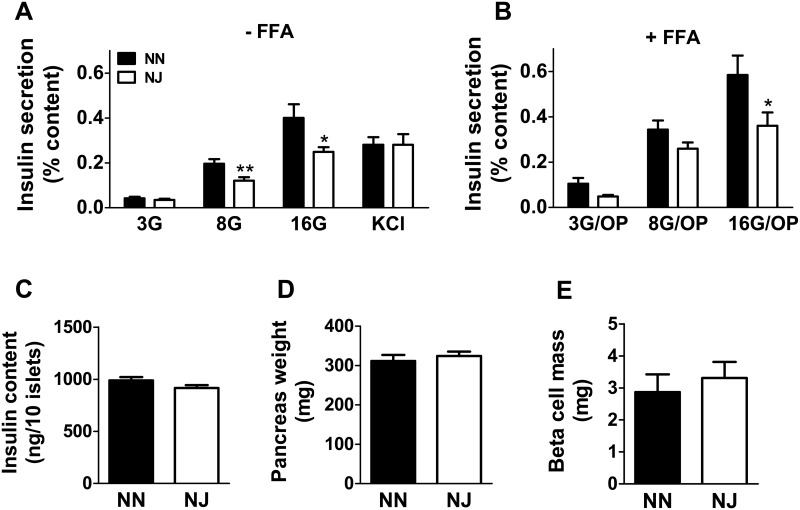
Insulin secretion in isolated islets from HFD-fed NN or NJ mice. Insulin secretion at 3 mM glucose plus 35 mM KCl and at 3, 8 and 16 mM glucose in the absence (3G, 8G and 16G) **(A)** or the presence **(B)** of palmitate/oleate (OP; 0.15mM each) (3G/OP, 8G/OP and 16G/OP) (n = 4–5 mice, 3–4 replicates/mouse). Insulin release was normalized for the total islet insulin content. Insulin content/10 islets **(C)**, pancreas weight **(D)** and beta-cell mass **(E)** of HFD-fed NN or NJ mice. Results are means ± SEM of 2–3 independent experiments. *p<0.05 and **p<0.01 compared to NN mice (Student’s t-test).

We further examined if the reduced insulin secretion observed *in vivo* in NJ mice compared to NN mice is due to an inherent defect in islets. Isolated pancreatic islets from HFD-fed mice were used to measure insulin secretion in response to glucose, fatty acids or 35 mM KCl. Consistent with the *in vivo* results, glucose-stimulated insulin secretion in NJ mice was lower than in islets from NN mice at 8 and 16 mM glucose (Fig 3A). In the presence of fatty acids, insulin secretion from NJ islets was significantly reduced in response to 16 mM glucose and a trend was observed at 8 mM glucose (p value = 0.08) (Fig 3B). KCl-induced insulin secretion was not altered in islets from NJ mice, compared to NN islets. There were no differences in islet insulin content (Fig 3C), pancreas weight (Fig 3D), and β-cell mass (Fig 3E) in NJ versus NN male mice. Thus, the lower glucose-induced insulin secretion response in NJ mice is not due to a lower islet insulin content, β-cell mass and pancreas weight or defective exocytosis process *per se* but is likely due to a defective metabolic signaling for secretion.

## Discussion

Metabolic and other studies often require the use of tissue-specific gene deleted mice to ascertain the function of a particular gene in a given tissue and many such studies have been reported over the last several years. However, in many of these studies, the mice used were not matched precisely for their background, as genotype-related differences were often ignored. We now report that the mixed genotype background mice that are most frequently used (C57BL/6NJ) show different phenotype, particularly under diet-induced obesity conditions, than their littermate controls of C57BL/6NN background and that grouping of mice with mismatched genotypes can lead to misleading conclusions.

The profound differences in insulinemia of HFD fed and fasted mice of NN and NJ genotypes are clearly indicative of an altered fuel-responsiveness of NJ islets to secrete insulin. This became evident following oral glucose tolerance test, which showed lower glucose-stimulated insulin secretion in HFD-fed NJ mice. However, the difference in insulinemia is not reflected in the corresponding glycemia of these mice, although there was a trend for NJ mice on HFD to be more glucose intolerant than the NN mice. We and others previously showed that fed insulin levels are lower in JJ mice [13,18], similar to what we noticed here in NJ mice, as compared to NN mice, indicating that genetic differences that contribute to the reduced insulin levels in NJ mice likely arise from the JJ substrain. Similarly, our earlier study [13] indicated a lack of difference in glucose tolerance following oral glucose challenge between NN and JJ mice under ND. The absence of insulin secretion defect and glucose intolerance on NJ mice on ND could be related to the use of OGTT in the present study. Indeed, we previously showed impaired GSIS in JJ mice when glucose is administered intravenously but not in response to oral glucose. Thus, a defective insulin secretion might be observed after intravenously glucose administration in NJ mice on ND condition. Moreover, it appears that HFD-induced metabolic stress precipitates the glucose intolerance of JJ strain, which is also noticed in NJ mixed background mice.

Although NJ mice showed reduced glucose-induced insulin secretion but similar glycemia compared to NN mice, this was not due to enhanced insulin sensitivity and also not because of altered β-cell mass or islet insulin content, as previously reported for JJ versus NN mice [13,17]. We considered the possibility that elevated expression of insulin degradation enzyme, coming from C57BL/6J genetic background [28] may cause the lowered insulinemia in NJ mixed background mice. However, it is unlikely that increased insulin clearance contributed to the lower insulinemia levels in NJ mice as JJ mice do not have altered insulin clearance in comparison to NN mice [13]. The reduced insulin secretion response seems to be because of defective glucose signaling for insulin secretion seen in islets from HFD-fed NJ mice. Proximal rather than distal processes to [Ca^2+^] rises might be defective in NJ mouse islets as there was no change in secretion response to a depolarizing concentration of KCl. These results are in line with previous reports indicating that isolated islets from JJ mice have a normal response to the potassium channel blocker tolbutamide [12,15].

A deletion mutation in NNT gene, seen in C57BL/6J mice, has been shown to affect insulin secretion and transgenic expression of NNT was able to rescue and restore normal insulin secretion in these mice [15]. Moreover, NNT catalyzes the reversible reduction of NADP^+^ by NADH and the conversion of NADH into NAD^+^. NAD(P)H/NAD(P)^+^ ratios are known to be involved in the control of insulin secretion [33] and changes in these ratios due to NNT mutation can explain at least in part the secretory defect in JJ or NJ mice. However, other genotypic differences between JJ and NN mice such as a retrotransposon insertion into an intron of *Rptor*, coding for Raptor, a key regulator of mTORC1 signaling [16] and also other mutations [2] might also possibly contribute to the observed insulin secretion alterations in the NJ mice.

Besides, different experimental conditions, animal suppliers (even if they have the same nominal genotype) and diets may also have an influence on the outcome of experiments. Thus, while we did not notice any differences in body weight and food intake under a normal or a high-fat diet between NN vs NJ mice as previously shown by Wong and colleagues in JJ mice [17], other studies reported some differences [14,19–21,34].

## Conclusion

Altogether, our results show altered insulin secretion in HFD-fed C57BL/6NJ mice, showing for the first time that metabolic parameters are affected differently in mixed C57BL/6 substrain (NJ) mice as compared to NN mice. These results highlight the importance of selecting mice with a pure C57BL/6NN (or JJ) genetic background with appropriate littermate control mice for any metabolic studies. Also, since NJ mice have different metabolic responses compared to NN mice at least under metabolic stress, removing NJ mice from studies may improve reproducibility and, therefore, the overall use of the animals. Although our observations limit this conclusion to metabolic studies, it is better to use mice with pure genotype background for any studies that warrant the use of tissue specific gene deletion to prevent the possibility of erroneous conclusions.
